# Quality of basic life support when using different commercially available public access defibrillators

**DOI:** 10.1186/s13049-015-0123-1

**Published:** 2015-06-21

**Authors:** Michael P. Müller, Cynthia Poenicke, Maxi Kurth, Torsten Richter, Thea Koch, Carolin Eisold, Adrian Pfältzer, Axel R. Heller

**Affiliations:** Department of Anaesthesiology and Intensive Care Medicine, University Hospital, Technische Universität Dresden, Dresden, Germany

**Keywords:** Defibrillation, Resuscitation, Chest Compression Resuscitation

## Abstract

**Background:**

Basic life support (BLS) guidelines focus on chest compressions with a minimal no-flow fraction (NFF), early defibrillation, and a short perishock pause. By using an automated external defibrillator (AED) lay persons are guided through the process of attaching electrodes and initiating defibrillation. It is unclear, however, to what extent the voice instructions given by the AED might influence the quality of initial resuscitation.

**Methods:**

Using a patient simulator, 8 different commercially available AEDs were evaluated within two different BLS scenarios (ventricular fibrillation vs. asystole). A BLS certified instructor acted according to the current European Resuscitation Council 2010 Guidelines and followed all of the AED voice prompts. In a second set of scenarios, the rescuer anticipated the appropriate actions and started already before the AED stopped speaking. A BLS scenario without AED served as the control. All scenarios were run three times.

**Results:**

The time until the first chest compression was 25 ± 2 seconds without the AED and ranged from 50 ± 3 to 148 ± 13 seconds with the AED depending on the model used. The NFF was .26 ± .01 without the AED and between .37 ± .01 and .72 ± .01 when an AED was used. The perishock pause ranged from 12 ± 0 to 46 ± 0 seconds. The optimized sequence of actions reduced the NFF, which ranged now from .32 ± .01 to .41 ± .01, and the perishock pause ranging from 1 ± 1 to 19 ± 1 seconds.

**Conclusions:**

Voice prompts given by commercially available AED merely meet the requirements of current evidence in basic life support. Furthermore, there is a significant difference between devices with regard to time until the first chest compression, perishock pause, no-flow fraction and other objective measures of the quality of BLS. However, the BLS quality may be improved with optimized handling of the AED. Thus, rescuers should be trained on the respective AED devices, and manufacturers should expend more effort in improving user guidance to shorten the NFF and perishock pause.

**Electronic supplementary material:**

The online version of this article (doi:10.1186/s13049-015-0123-1) contains supplementary material, which is available to authorized users.

## Introduction

The survival rate after out-of-hospital cardiac arrest (OHCA) is below 10 %, despite considerable efforts on the part of emergency medical services and hospitals to optimize treatment [1]. The key problem is that in cases where no lay rescuers initiate basic life support measures, blood flow to the brain is restored only when professional rescuers start chest compressions, which is often too late for recovery without serious sequelae. The current American Heart Association and European Resuscitation Council guidelines for cardiopulmonary resuscitation (CPR) outline immediate recognition, early CPR, and rapid defibrillation as the key elements of the chain of survival [2,3]. Furthermore, the 2010 update of the CPR guidelines was designed to ensure high quality chest compressions and minimize interruptions to maximize organ perfusion*.*

Automated external defibrillators (AED) reliably recognize shockable cardiac rhythms and allow defibrillation to be performed by non-physicians. Furthermore, the time from collapse to defibrillation determines survival in patients with OHCA; each delay of one minute increases mortality by 10 % [4]. Thus, efforts have been increasingly undertaken in the past few years to make AEDs available to the public, especially in high-traffic public areas. By decreasing the time to the first defibrillation, the survival rate after OHCA has increased up to 74 % [5]. Many different AEDs are commercially available, and modern devices defibrillate with biphasic waveforms, resulting in high conversion rates. AEDs seem to be perfect “resuscitation devices” for lay rescuers, as they not only analyse the rhythm and shock the patient if indicated, but they also provide essential instructions regarding the BLS algorithm. The AED provides audible instructions to activate the emergency medical system (EMS), check for vital signs, and initiate chest compressions. However, as early as 10 years ago, Fleischhackl studied how lay people operate an AED and found alarming results; the time to the first shock and the proportion of study volunteers who started chest compressions after being prompted by the AED varied widely [6].

To our knowledge, no previous study has investigated interruptions of chest compressions during BLS when an AED is used. The objective of this study was to evaluate the quality of BLS when an AED is used with a special focus on the no-flow fraction and the perishock pause.

## Methods

This study complies with the Declaration of Helsinki, and the locally appointed ethics committee approved the study protocol (EK 127042012). Two standard test scenarios were scripted using a Code Blue III Advanced Life Support manikin (Gaumard Scientific, Miami, FL/ USA). The manikin has conductive skin regions allowing defibrillation with real AEDs through the standard self-adhesive pads. The simulator can be connected to a laptop computer, and the Gaumard software (Gaumard UI-1.40.18.0) records chest compressions, ventilations, and defibrillation.

Eight AEDs were available for the study: Heartsave PAD (Metrax, Rottweil, Germany), Philips HS1 (Philips Medical Systems, Bothell, WA, USA), Lifeline VIEW AED (Defibtech, Guilford, CT, USA), Lifepak CR Plus (Medtronic Physio-Control, Redmond, WA, USA), PowerHeart G5 (Cardiac Science, Bothell, WA, USA), Fred Easy Life (Schiller AG, Baar, Switzerland), AED Plus (Zoll Medical Corporation, Chelmsford, MA, USA), and Cardiolife AED 2100 (Nihon Kohden Corporation, Tokio, Japan). All AEDs were provided by the manufacturers, except for the AED Plus. The language of all AEDs were set to German. The manufacturers were asked to provide a device that is used as a public access defibrillator. Each of the AEDs was evaluated in pre-scripted basic life support scenarios with an adult patient in persistent ventricular fibrillation (VF, scenario 1) or asystole (scenario 2). Each AED was tested three times in both scenarios. The scenario duration was 5 minutes. One BLS instructor (MK) performed single-rescuer BLS using an AED and following the voice prompts. BLS was performed according to the 2010 ERC guidelines, with a ratio of 30 chest compressions to 2 ventilations. The respective AED was switched on immediately at the start of each scenario. The BLS instructor followed all voice prompts of the AED exactly as provided. The rescuer listened to each voice prompt and started the appropriate action immediately after the AED finished speaking.

All AEDs were tested again in a second set of scenarios (a total of three times each in scenarios 1 and 2). However, the BLS caregiver followed the optimal sequence of actions, with minimal interruptions in chest compressions. This was done by starting the appropriate action as early as possible during the AED voice prompt*.* Some actions can be initiated before the AED has started that particular instruction; for example, chest compressions were resumed immediately after each shock.

A third scenario, 5 minutes in duration, was run without the use of an AED to calculate the difference in the no-flow fraction between BLS with and without the use of an AED. This scenario was also repeated three times.

During all scenarios with the use of an AED, the following values and time intervals were measured:

- Time until first chest compression
- Time until the first rhythm analysis was completed
- Duration of rhythm analysis
- Time until first shock (in scenarios with ventricular fibrillation)
- Time between first shock and the beginning of the second rhythm analysis (in scenarios with ventricular fibrillation) or time between completion of the first rhythm analysis and the beginning of the second rhythm analysis (in scenarios with asystole)
- Perishock pause: Time from last chest compression (CC) before a shock until the first chest compression after the respective shock (in scenarios with ventricular fibrillation)
- No-flow fraction (NFF): Fraction expressed as a quotient of the no-flow time and the total time without spontaneous circulation.

The mean and standard deviation were calculated for all parameters for each AED.

### Data analysis and statistics

All data were analysed using IBM SPSS software (Version 21.0.0.0, Armonk, NY). After ensuring homogeneity of variances by the Levene test, univariate ANOVA was used for repeated between-AED model analyses and was followed by Sidak post-hoc testing and multiple comparison alpha adjustment.

Within each AED model, a paired t-test was used to compare the effects of performing the optimal sequence of actions compared to standard AED voice-prompt-guided procedures.

Due to the strongly statistically significant difference between the AED models as shown in Tables 1, Table 2, Table, 3 and 4, only a lack of significance is indicated. If not otherwise indicated, the between AED-model comparison of the respective parameters is statistically significant at a p-value of <0.01.

**Table 1:** BLS with the use of an AED in scenarios of persistent ventricular fibrillationThe duration of the scenario was 300 seconds

	Time until 1st chest compression [seconds]	Time until completion of 1st rhythm analysis [seconds]	Duration of rhythm analysis [seconds]	Time until 1st shock [seconds]	Interval between 1st shock and 2nd rhythm analysis [seconds]	Perishock pause [seconds]	No-flow fraction
(A) Heartsave PAD	50 ± 3 ^I^	47 ± 3^D,I^	13 ± 1^B,C,D,F,G^	63 ± 4^D^	138 ± 0^C^	12 ± 0^+^	0.51 ± 0.01^C,E,G,+^
(B) Philips HS1^§^	125 ± 2	77 ± 3^C,G,H^	13 ± 2^A,C,D,E,F,G,H^	88 ± 2^C^	194 ± 3	44 ± 4^G^	0.72 ± 0.01^F^
(C) Philips HS1^*^	103 ± 1^G,+^	77 ± 3^B,G,H,+^	10 ± 1^A,B,D,E,F,H,I^	90 ± 2^B,+^	140 ± 1^A^	29 ± 1^E,H,+^	0.52 ± 0.01^A,G,+^
(D) Lifeline VIEW	68 ± 3^+^	48 ± 2^A,I,+^	11 ± 1^A,B,C,E,F,H,I^	56 ± 3^A,I,+^	153 ± 3	34 ± 2^E,F,+^	0.61 ± 0.03^+^
(E) Lifepak CR Plus	81 ± 1^H,+^	63 ± 1 ^H,+^	10 ± 1^B,C,D,F,H,I^	75 ± 1^G,H,+^	125 ± 1^G,H,I^	31 ± 1^C,D,+^	0.48 ± 0^A,G,H,+^
(F) PowerHeart G5	147 ± 1^+^	93 ± 1^+^	11 ± 1^A,B,C,D,E,H^	101 ± 1^+^	183 ± 1	38 ± 1^D,+^	0.71 ± 0^B,+^
(G) Fred Easy Life	105 ± 1^C,+^	74 ± 1^B,C,H,+^	15 ± 0^A,B^	80 ± 1^E,H,+^	124 ± 1^E,H,I^	46 ± 0^B,+^	0.50 ± 0^A,C,E,+^
(H) AED Plus	81 ± 3^E,+^	71 ± 2^B,C,E,G,+^	9 ± 1^B,C,D,E,F,I^	77 ± 2^E,G,+^	121 ± 1^E,G,I^	25 ± 1^C,I,+^	0.45 ± 0.01^E^
(I) Cardiolife AED 2100	55 ± 1^A,+^	41 ± 2^A,D,+^	7 ± 1^C,D,H,I^	48 ± 2^+^	126 ± 1^E,G,H^	21 ± 2^H^	0.37 ± 0.01
(K) BLS without AED	25 ± 2						0.26 ± 0.01

Values are the mean ± standard deviation. The Philips HS1 asks the rescuer to press an “info button” for detailed information about chest compressions and ventilations after each shock. The scenario was run 3 times when pressing the info button^§^ and 3 times without pressing the button^*^. A lack of statistical significance (p > 0.01) in comparison with a specific model is indicated by the identifying letter (A) – (I). ^+^p < 0.01 against the respective parameter in the scenarios with an optimized sequence of actions

**Table 2:** BLS with the use of an AED in scenarios of persistent asystole

	Time until 1st chest compression [seconds]	Time until completion of 1st rhythm analysis [seconds]	Duration of rhythm analysis [seconds]	Interval between completion of 1st rhythm analysis and start of 2nd rhythm analysis [seconds]	No-flow fraction
(A) Heartsave PAD	58^+^ ± 2 ^D,E,H,I,+^	50 ± 3^D,H,I^	20 ± 1	140 ± 1^C^	0.52 ± 0.03^C^
(B) Philips HS1^§^	112 ± 2^G^	77 ± 3^C,G^	11 ± 1^C,D,E,I^	200 ± 1	0.71 ± 0^F^
(C) Philips HS1^*^	92 ± 2^G,+^	78 ± 2^B,G,+^	12 ± 0^B,D,E,I^	140 ± 0^A^	0.50 ± 0^A,+^
(D) Lifeline VIEW	64 ± 1^A,E,H,I,+^	53 ± 2^A,H^	12 ± 1^B,C,F,I^	154 ± 1	0.58 ± 0.01^+^
(E) Lifepak CR Plus	67 ± 1 ^A,D,H,I,+^	61 ± 1^+^	10 ± 1^B,C,H,I^	125 ± 0^G,H,I^	0.42 ± 0.01^G,H,I^
(F) PowerHeart G5	148 ± 13^+^	96 ± 2^+^	15 ± 1^D,G^	183 ± 1	0.72 ± 0^B,+^
(G) Fred Easy Life	97 ± 1 ^B,C,+^	74 ± 1^B,C,+^	15 ± 0^F^	123 ± 1^E^	0.45 ± 0^E,H,+^
(H) AED Plus	57 ± 1^A,D,E,I,+^	53 ± 2^A,D,+^	8 ± 0^E,I^	126 ± 1^E,I^	0.41 ± 0.01^E,G,I,+^
(I) Cardiolife AED 2100	52 ± 1^A,D,E,H,+^	45 ± 1^A,+^	10 ± 0^B,C,D,E,H^	127 ± 0^E,H^	0.40 ± 0.01^E,H^
(K) BLS without AED	25 ± 2				0.26 ± 0.01

The duration of the scenario was 300 seconds. Values are the mean ± standard deviation. The Philips HS1 asks the rescuer to press an “info button” for detailed information about chest compressions and ventilations after each rhythm analysis. The scenario was run 3 times when pressing the info button^§^ and 3 times without pressing the button^*^. A lack of statistical significance (p > 0.01) in comparison with a specific model is indicated by the identifying letter (A) – (I). ^+^p < 0.01 against the respective parameter in the scenarios with an optimized sequence of actions.

**Table 3:** BLS with the use of an AED in scenarios of persistent ventricular fibrillation: optimized sequence of actionsThe duration of the scenario was 300 seconds

	Time until 1st chest compression [seconds]	Time until completion of 1st rhythm analysis [seconds]	Duration of rhythm analysis [seconds]	Time until 1st shock [seconds]	Interval between 1st shock and start of 2nd rhythm analysis [seconds]	Perishock pause [seconds]	No-flow fraction
(A) Heartsave PAD	35 ± 2^B,C,E,G^	34 ± 2 ^B,C,E^	14 ± 1 ^B,C,E,F^	47 ± 2 ^F^	137 ± 1 ^B^	3 ± 1 ^D,+^	0.36 ± 0.01^B,C,D,E,F,G,H,+^
(B) Philips HS1	31 ± 2^A,D,E,G,H,K,+^	28 ± 2 ^A,D,E,G,H,+^	14 ± 2 ^A,C,E,F,G^	30 ± 2 ^E,G,H,+^	140 ± 0 ^A^	10 ± 1 ^E,H,+^	0.34 ± 0.01^A,C,D,E,F,G,H,+^
(C) Lifeline VIEW	41 ± 1 ^A,+^	35 ± 1 ^A,E,+^	11 ± 2 ^A,B,D,E,G,H^	39 ± 1 ^D,+^	154 ± 1	18 ± 1 ^F,G,+^	0.36 ± 0.01^A,B,D,E,F,G,H,+^
(D) Lifepak CR Plus	28 ± 2^B,E,G,H,K,+^	27 ± 2 ^B,E,G,H,+^	10 ± 1 ^B,C,E,G,H^	38 ± 3 ^C,E,+^	125 ± 1 ^F,G,H^	1 ± 1 ^A,+^	0.32 ± 0.01^A,B,C,E,F,H,+^
(E) PowerHeart G5	34 ± 1^A,B,D,G,H,+^	30 ± 2 ^A,B,C,D,G,+^	12 ± 1 ^A,B,C,D,F,G,H^	32 ± 1 ^B,D,G,H,+^	185 ± 2	13 ± 2 ^B,G,H,+^	0.33 ± 0.01^A,B,C,D,F,G,H,+^
(F) Fred Easy Life	17 ± 2^+^	49 ± 1^+^	15 ± 1 ^A,B,E^	51 ± 2 ^A,+^	124 ± 1 ^D,G,H^	19 ± 1 ^C,G,+^	0.35 ± 0.01^A,B,C,D,E,G,H,+^
(G) AED Plus	33 ± 2^A,B,D,E,H,+^	27 ± 2 ^B,D,E,H,+^	10 ± 1 ^B,C,D,E,H^	31 ± 2 ^B,E,H,+^	125 ± 1 ^D,F,H^	18 ± 1 ^C,E,F,+^	0.37 ± 0.02^A,B,C,E,F,H^
(H) Cardiolife AED 2100	27 ± 1^B,D,E,G,K,+^	23 ± 0 ^B,D,G,+^	8 ± 1 ^C,D,E,G^	26 ± 1 ^B,E,G,+^	127 ± 2 ^D,F,G^	11 ± 1 ^B,E^	0.35 ± 0.02^A,B,C,D,E,F,G^
(K) BLS without AED	25 ± 2^B,D,H^						0.26 ± 0.01

Values are the mean ± standard deviation. A lack of statistical significance (p > 0.01) in comparison with a specific model is indicated by the identifying letter (A) – (I). ^+^p < 0.01 against the respective parameter in the scenarios with a standard sequence of actions

**Table 4:** BLS with the use of an AED in scenarios with persistent asystole: optimized sequence of actions

	Time until 1st chest compression [seconds]	Time until completion of 1st rhythm analysis [seconds]	Duration of rhythm analysis [seconds]	Interval between completion of 1st rhythm analysis and start of 2nd rhythm analysis [seconds]	No-flow fraction
(A) Heartsave PAD	40 ± 2^C,E,+^	39 ± 3	19 ± 2	140 ± 1^B^	0.41 ± 0.01
(B) Philips HS1	26 ± 1^D,G,H,K,+^	24 ± 1^D,G,H,+^	10 ± 1^C,D,G,H^	142 ± 1^A^	0.33 ± 0.02^C,D,E,F,G,H,+^
(C) Lifeline VIEW	33 ± 2^A,D,E,G,H,+^	32 ± 2^D,E,+^	12 ± 1^B,D,E,F^	153 ± 0	0.36 ± 0.02^B,D,E,F,G,H,+^
(D) Lifepak CR Plus	28 ± 2^B,C,E,G,H,K,+^	26 ± 1^B,C,E,G,H,+^	10 ± 1^B,C,G,H^	125 ± 0^G^	0.33 ± 0.01^B,C,E,F,G,H^
(E) PowerHeart G5	34 ± 0^A,C,D,G,+^	32 ± 1^C,D,+^	14 ± 1^C,F^	184 ± 1	0.34 ± 0.01^B,C,D,F,G,H,+^
(F) Fred Easy Life	16 ± 2^+^	48 ± 1^+^	15 ± 1^C,E^	122 ± 1^G^	0.33 ± 0.01^B,C,D,E,G,H,+^
(G) AED Plus	27 ± 2^B,C,D,E,H,K,+^	26 ± 1^B,D,H,+^	9 ± 0^B,D,H^	125 ± 1^D,F^	0.35 ± 0^B,C,D,E,F,H,+^
(H) Cardiolife AED 2100	27 ± 3^B,C,D,G,K,+^	25 ± 3^B,D,G,+^	9 ± 0^B,D,G^	128 ± 1	0.35 ± 0^B,C,D,E,F,G^
(K) BLS without AED	25 ± 2^B,D,G,H^				0.26 ± 0.01

The duration of the scenario was 300 seconds. Values are the mean ± standard deviation. A lack of statistical significance (p > 0.01) in comparison with a specific model is indicated by the identifying letter (A) – (I). ^+^p < 0.01 against the respective parameter in the scenarios with a standard sequence of actions.

## Results

Data for BLS with an AED when the AED voice prompts were strictly followed are shown in Table 1 for VF and in Table 2 for asystole. Table 3 (VF) and Table 4 (asystole) present the data from the AED scenarios with an optimized sequence of actions during BLS. An English translation of the voice prompts in a scenario with shockable rhythm is provided for all AEDs that were evaluated (Additional file 1).

In the scenarios with persistent ventricular fibrillation and without an AED, the rescuer started chest compressions after 25 ± 2 seconds. When using an AED and following the voice prompts, the mean time until the first chest compression ranged from 50 ± 3 seconds to 148 ± 13 seconds in the first set of scenarios (the action was started after the AED finished speaking). In the second set (the optimized sequence of actions), the interval to the start of chest compressions ranged from 16 ± 2 to 41 ± 1 seconds.

The no-flow fraction during BLS without the use of an AED was 0.26 ± 0.01. However, the use of an AED increased the NFF in all scenarios: in the first set of scenarios, the NFF ranged between 0.37 ± 0.01 and 0.72 ± 0.01 (VF) and between 0.40 ± 0.01 and 0.72 ± 0 (asystole). In the second set of scenarios (the optimized sequence), the variability in NFF decreased to 0.32 ± 0.01 – 0.37 ± 0.02 for VF and 0.33 ± 0.01 – 0.41 ± 0.01 for asystole, respectively. The differences between the devices were statistically significant, except for three models of VF and one model of asystole. However, the NFF still was significantly higher with all devices compared to BLS without the use of an AED (p < 0.01).

In the first set of scenarios, the shortest value for the perishock pause was 12 ± 0 seconds, whereas the longest pause was 46 ± 0 seconds. In the scenarios with an optimized sequence of actions, the perishock pause was shorter, ranging from 1 ± 1 to 19 ± 1.

## Discussion

This is the first study to investigate the no-flow fraction, perishock pause, and other objective quality parameters during BLS using eight different available public access defibrillators in a simulator setting.

As expected, our study found higher NFF values in BLS scenarios that used an AED compared to those that did not. Furthermore, we found extremely high NFF values in these scenarios with a high variability between the different models. In all cases, the perishock pause was always much longer than advised by the current resuscitation guidelines. However, when the rescuer optimized the sequence of actions and acted immediately before or after the voice prompt started (Tables 2 and 4), the NFF and the perishock pause both were significantly reduced, with minor variability.

The chest compression fraction has been identified as a cardiac resuscitation quality parameter that is associated with the survival rate [7,8]. A small study from the PAD trial evaluated the quality of BLS and found very high values for NFF (0.52 to .83) when lay rescuers performed BLS and used an AED [9]. Two studies have shown that chest compressions during prehospital life support are interrupted in approximately half of the time [10,11]. The current international resuscitation guidelines demand high quality chest compressions with minimal interruptions. Well trained teams can provide life support with NFFs as low as 0.24 [12]. In these cases, a defibrillator capable of manual defibrillation was used. However, an observational study showed that the NFF during in-hospital BLS with the use of an AED can be as low as 0.30 when nurses repetitively perform the BLS/ AED algorithm in a BLS training course [13].

The NFF during scenario 2 (asystole) was comparable to that of scenario 1 and was significantly higher than the value for BLS without an AED for all AEDs. Recent studies have found that only between 24 and 40 % of patients with OHCA are in a shockable rhythm when the ambulance arrives [14]. At the time of collapse, the proportion of patients who are in a shockable rhythm is higher, but there is still a considerable number of patients who are in a non-shockable rhythm. For those patients, the use of an AED resulting in a high NFF is harmful and may increase mortality.

The NFF was lower for six devices (VF) and 5 devices (asystole) during the scenarios with an optimized sequence of actions. The NFF was similar in all devices and was below .40, except for one AED in asystole. This set of scenarios can be compared with trained lay rescuers or healthcare professionals after practice with a specific AED. The first set of scenarios involves untrained lay rescuers using a public access defibrillator while strictly adhering to the guiding voice prompts. It is concerning that the use of commonly available AEDs, which are designed to be used by lay rescuers, might lead to poor quality life support, with chest compressions being delivered less than 30 % of the time. The wide difference between the standard use and the optimized sequence of actions implies that the human-machine interface of the devices is a major problem; manufacturers should expend considerably more effort on the development of optimal user guidance to ensure the best possible performance, even when used by lay rescuers who have never before observed a patient in cardiac arrest and who perhaps have never received BLS training.

### Perishock pause

Survival to hospital discharge is substantially more likely when the first documented rhythm is shockable rather than nonshockable [15]. When a defibrillator is used, the perishock pause has been identified as an independent predictor of survival [16]. A substantial delay in providing chest compressions has been observed in the post-shock period with AED-equipped first responders [17]. As Cheskes et al. reported, a pre-shock pause of ≥ 20 seconds and a perishock pause of ≥ 40 seconds are predictors of worse survival after shockable cardiac arrest. Based on a log-linear model, any five seconds increase in the perishock pause interval decreases the rate of patient survival to hospital discharge by 14 % [16].

Therefore, the international guidelines for resuscitation advise a perishock pause of less than 5 seconds. This recommendation was not fulfilled in any of the AEDs tested in the first set of scenarios with strict voice prompt adherence. We noted that some AEDs do not permit chest compressions during the charging period, resulting in an almost fourfold variation in the perishock pause between the different devices. This variation in duration may be responsible for the variable success in patient resuscitation. In 2004, Snyder et al. alluded to the problem of the AED-determined variation in hands-off times[18]. However, the manufacturers may have implemented an algorithm, which does not prompt the rescuer to provide chest compressions for some seconds during charge and then take hands off for the shock as this may confuse lay persons. We need further studies to find out whether real lay rescuers are able to follow such an algorithm with minimised perishock pause.

The perishock pause was reduced in nearly all of the tested AEDs when the optimized sequence of actions (in the second set of scenarios) was used. Two devices met the demands of the current guidelines, implying that there is significant potential for improving user guidance. The necessary changes are small and include optimization of the device algorithms and voice prompts. Determining the best practices for AED voice prompting will be the subject of a follow-up analysis of the present dataset.

### Time until first chest compression

A lone lay rescuer following the AHA guidelines for BLS would call an ambulance, fetch an AED (if available), and return to the patient to start life support [2]. However, when switching on the AED and following the voice prompts, the beginning of chest compressions is delayed for up to nearly 2.5 minutes. This stands in sharp contrast to what is taught in courses on cardiac resuscitation around the world: the check – call – compress paradigm, in which chest compressions are started as early as possible, and any unnecessary interruptions are avoided.

A retrospective analysis of OHCA cases found no benefit of bystander CPR for patients receiving the first shock between 1 and 5 minutes after collapse, whereas survival was much higher for patients receiving bystander CPR when the first shock was applied after more than 11 minutes [19]. However, even for the patients receiving the first shock within 5 minutes, this study does not answer the question whether shock first (and delayed start of chest compressions) or CPR first (and later application of the shock) is superior with regard to the survival rate. However, modern AED devices should guide the lay rescuer to soonest possible defibrillation, while assuring a short time until the first chest compression.

### Duration of rhythm analysis

The duration of rhythm analysis depends solely on the technical specifications of the respective device. This value varied by about 100 % between the devices. However, the absolute difference between the fastest and slowest analysis duration was only 8 seconds. One potential future improvement is hands-on rhythm analysis during chest compressions. Making a serious effort to develop optimized devices may lead to shorter time intervals. However, a much larger effect on shortening the NFF among layperson rescuers could be achieved by inventing devices with improved usability and voice prompts.

### Wide variation of CPR quality between different AED models

When a lay rescuer provides BLS and uses an AED model with which he or she is not familiar, the CPR quality depends critically on the AED device. We found extensive variations in important parameters such as the NFF and the perishock pause. Most clinical studies investigating the effects of the use of AED were not stratified by the AED model used. Future studies, especially large resuscitation registries, should always consider the defibrillator model. Even the firmware version might provide important additional information, as different versions may differ in terms of user guidance and thus lead to variations in the quality of BLS.

In this study, a trained person used the AEDs. However, lay rescuers use these machines in real cardiac arrest situations. As they are under high stress and may be afraid of using an AED, the voice prompts should be clear and precise. Some models provide more detailed voice prompts than others (see Additional file 1). We do not know whether more details result in better performance and less mistakes when the AED is used by untrained real lay rescuers and whether this may compensate the longer and more frequent interruptions. The intention of very precise instructions may be to assure a shock when used by a heterogeneous group of lay rescuers. However, if a lone rescuer places an emergency call and fetches an AED before returning to the patient, 2 minutes or more may already have been expired. At this time, soonest beginning of chest compressions is important and this should be taken into account when developing AEDs and when elaborating user guidance. Further research should be done to study the quality of BLS when lay rescuers use AEDs.

### Limitations

This study was performed in a simulator centre, and a BLS instructor evaluated the devices. The quality of BLS in real cases may vary from that in this study.

With some AEDs, lay rescuers may not understand some voice prompts or they could experience usability problems that could not been found in the present study but may result in even worse BLS performance.

## Conclusions

Our study presents concerning results regarding the quality of BLS when using an AED and following all voice prompts. We found a broad variation in the parameters, which were evaluated in the study, including the time until the first chest compression, the no-flow fraction, and the perishock pause. The manufacturers should take these results into account when developing the next generation of AEDs to be used by layperson rescuers: The protocol of the respective device should meet the best available standard as a high quality of BLS is of utmost importance for optimal survival rates.

## Additional file

Additional file 1: Table S1. Voice prompts of the AEDs in a scenario with shockable rhythm.
