# Supplementary material for: Quality of basic life support when using different commercially available public access defibrillators
Source: Scand J Trauma Resusc Emerg Med. 2015 Jun 21;23:48. doi: 10.1186/s13049-015-0123-1 (PMC4475613; doi:10.1186/s13049-015-0123-1)
Supplement: Supplementary file 1 [file 13049_2015_123_MOESM1_ESM.docx]

Table 5a: Voice prompts of the AEDs in a scenario with shockable rhythm.

| Heartsave PAD | Philips HS1 | Lifeline View | Lifepak CR Plus |
| --- | --- | --- | --- |
| Before rhythm analysis |
| „Adult mode.“ „Place emergency call.“ „Place the electrodes on the bared chest one after the other.“ (repeated until electrodes are placed) “Do not touch the patient. Rhythm analysis.” | „Remove all clothes from the patient`s chest. If necessary, cut the clothes.” “After baring the chest, remove the white protective foil and take out the electrodes.” “Take notice of the illustrations depicted on the electrodes.” “Remove the yellow foil from one electrode. Place the electrode as illustrated. Press it firmly on the bared chest.” “After this, place the 2nd electrode as illustrated. Remove the yellow foil from the 2nd electrode. Place it as illustrated. Press it firmly on the bared chest.” | „Call for help.“ „Follow the instructions for placing the electrodes.” “Remove the clothes from the patient`s chest.” “Take the electrodes out of the package on the back side of the device.” “Open the package with the electrodes.” “Remove the electrodes from the blue foil.” “Place the electrodes on the bare chest as illustrated.” “Stop all movements. Analysis of the cardiac rhythm. Do not touch the patient.” | „Attention! Follow the instructions of the device.“ “Arrange for emergency call.” “Remove all clothes from the patient`s chest.” “Pull the red grip to open the package and take out the electrodes.” “Take notice of the illustrations on the adhesive electrodes.” “Remove one electrode from the blue foil.” “Stick the adhesive electrode firmly on the bare chest as illustrated.” “Press the electrode firmly on the chest.” “Remove the other electrode from the blue foil.” “Stick the adhesive electrode firmly on the bare chest as illustrated.” “Press the electrode firmly on the chest.” “Attention. Do not touch the patient.” “Cardiac rhythm is being analyzed.” |
| Analysis |
| “Shock recommended.” “Chest compressions.” (15 beeps) “Step back from the patient.” “Push the blinking button now.” | “Do not touch the patient. Cardiac rhythm is being analyzed.” “Do not touch the patient. Shock recommended.” “Stay away from the patient by all means.” “Push the orange blinking button now.” | “Step back from the patient. Shock recommended.” “Charging in progress.”  (tone) „Push the blinking shock button.“ | “Ready. Shock is being prepared.” “Attention. Nobody must touch the patient now.” “Push the blinking button.” |
| Shock |
| “Cardiopulmonary resuscitation. 30 chest compressions.” (metronome) „2 ventilations.“ (repeated 5 times) „Do not touch the patient. Cardiac rhythm analysis.” (3 times repeated) “Shock recommended. Chest compressions.” (15 beeps) „Step back from the patient.” “Now push the blinking button.” “Cardiopulmonary resuscitation. 30 chest compressions.” (metronome) | “Shock applied.” “Call an ambulance.” “The patient can now be touched without any danger. Start chest compressions and ventilations.” “For further instructions concerning cardiopulmonary resuscitation, push the blue button.” §“Put one ball of the thumb in the middle of the chest. Put the second hand onto the first one. Push the chest down approximately 5 centimeters. Follow the rhythm of the beeps.” §“Close the patient`s nose. Tilt the head back. Lift the chin and ventilate. Ventilate. Ventilate. §“Go on performing chest compressions.” (metronome) “Stop chest compressions and ventilations. Do not touch the patient.” “Cardiac rhythm is being analyzed.” “Do not touch the patient. Shock recommended.” “Stay away from the patient by all means.” “Push the orange blinking button now.” “Shock applied.” “The patient can now be touched without danger. Start chest compressions and ventilations.” “For instructions concerning cardiopulmonary resuscitation, push the blue blinking button.” | “Shock 1 applied.” “You can touch the patient now without danger.” “Start cardiopulmonary resuscitation now.” “Perform chest compressions.” (metronome) “Perform ventilations. Ventilate. Ventilate.“ (Repetition of chest compressions and ventilations 5x) “Stop cardiopulmonary resuscitation. Stop it now.” “Do not touch the patient.” “Analysis of the cardiac rhythm. Do not touch the patient.” “Step back from the patient.“ “Shock recommended.” (tone)  „Push the blinking shock button.“ “Shock 2 applied.” “You can touch the patient now without danger.” “Start cardiopulmonary resuscitation.” | “Shock has been applied.” “Perform 30 chest compressions and 2 ventilations alternately.” “Continue with the patient care.” (repeated every 15 sec) “Attention. Do not touch the patient.” “Cardiac rhythm is being analyzed.” “Ready. Shock is being prepared.” “Energy is being charged.” “Attention. Nobody must touch the patient.” “Push the blinking button.” “Shock has been applied.” “Perform 30 chest compressions and 2 ventilations alternately.” |

Table 5b: Voice prompts of the AEDs in a scenario with shockable rhythm.

| Powerheart G5 | Fred easy Life | AED plus | Cardio Life AED 2100 |
| --- | --- | --- | --- |
| Before rhythm analysis |
| „Stay calm.“ | „Stay calm and follow the instructions of the AED.“ | „AED okay.“ | „Adult mode.“ |
| „Follow these instructions.“ | „Electrodes for adults.“ | „If the patient is unconscious and not breathing.” |
| “Call the emergency. Emergency call 112.” | „Stay calm.“ |
| “Remove all clothes from the patient`s chest.” |
| „Call 112 immediately.“ | „Check consciousness.“ |
| “Remove the square package from the AED.” |
| “If the patient is unconscious and not breathing normally, remove all clothes from the chest.” | „Make an emergency call.“ |
| “Open the package and take out the electrodes.” |
| „Bare the patient`s chest.“ | „Free airway. Check breathing.“ |
| “Remove the blue foil from the electrodes and place them on the right upper chest and on the left side of the body as illustrated.” (repeating until electrodes are placed) |
| „Place electrodes on bared chest.“ (repeating until electrodes are placed) |
| “Remove or cut the clothes.” | “If necessary, cut the clothes.” |
| “Don`t touch the patient. Analysis in progress.” |
| “Don`t touch the patient. Heart rhythm is being analyzed.” |
| “After baring the chest, take the white package from the top of the AED.” | “Open electrode package.” |
| “If necessary, plug the connector of the electrodes in the defibrillator.” |
| “Open the white package at the broken line and take out the electrodes.” |
| “Remove the first adhesive electrode from the protective foil and place it on the bared and dry chest as illustrated.” |
| “Remove one white electrode from the blue pad completely. Start at the strap in the corner.” |
| “Remove the second adhesive electrode from the protective foil and place it as illustrated.” |
| “Place the electrode without the blue foil on the bared chest of the patient exactly as illustrated on the electrodes.” |
| “Don`t touch the patient- |
| Heart rhythm is being analyzed.” |
| “This electrode can be placed in one of the two positions as illustrated on the electrodes.” | “Don`t touch the patient. Analysis in progress.” |
| “Then remove the second white electrode from the blue foil.” |
| “Place the second electrode exactly as illustrated in the other position.” |
| “Don`t touch the patient. Heart rhythm is being analyzed. Please wait. “ (repeating) |
|  |
|  |
|  |
|  |
| Analysis |
| “Shock recommended.” | “Shock recommended. Don`t touch the patient.” | “Shock recommended.” | “Don`t touch the patient. Shock recommended.” |
| “Don`t touch the patient. Push the red blinking button to administrate a shock.” | “Don`t touch the patient.” |
| “Don`t touch the patient.” (tone) |
| “Push the blinking button.” |
| “Push the orange button.” | “Push the blinking button.” |
| Shock |
| “Shock applied.” | “Shock applied.” | “Shock applied.” | “Shock applied” |
| “The patient can be touched now.” | “Now the patient can be touched again.” | “Start with resuscitation.” | “The patient can be touched again now.” |
| “Take the green square package from the top of the AED.” | “Put one ball of the thumb in the middle of the chest, then put the second hand onto the first one. Push strongly and keep the elbows stretched. Perform chest compression to the rhythm of the clock signal.” | “Go on performing resuscitation.” |
| “Start with the cardiopulmonary resuscitation.” |
| “Stop resuscitation. Stop resuscitation.” |
| “Go on performing cardiopulmonary resuscitation.” (repeated every 30sec) |
| “Open the green package and take out the HLW device.” | “Don`t touch the patient.” |
| “5 times left. (tone) Don`t touch the patient.” |
| “Analysis in progress.” |
| “Heart rhythm is being analyzed. Don`t touch the patient.” |
| “Put the HLW device between the nipples on the patient`s chest.” | “Don`t touch the patient. Analysis in progress.” |
| “Electric shock recommended.” |
| “Don`t touch the patient.” (tone) |
| “Put one ball of the thumb on the HLW device.” | (Announcement repeats after one minute, no ventilation, metronome signal without interruption.) | “Shock recommended.” | “Push the blinking button.” |
| “Don`t touch the patient.” | “Electric shock applied.” |
| “Push the blinking button.” | “Patient can be touched now.” |
| “Put the other ball of the thumb onto the first hand.” | “Don`t touch the patient. Heart rhythm is being analyzed.” | „Shock applied.“ |
| “Lean on the patient with stretched arms.” | “Don´t touch the patient. Analysis in progress.” |
| “Shock recommended. Don`t touch the patient.” |
| “Push and release strongly up to one third of the depth of the chest.” |
| “Push the orange button.” |
| “Then perform 30 fast compressions.” | “Shock applied.” |
| “The patient can be touched again.” |
| “Then 2 ventilations.” |
| “Start HLW.” |
| “Push. Push. Push.” (30 repeats) |
| (feedback, for example push stronger, release completely etc) |
| “Stop compressions. Ventilation. Ventilation.“ |
| „Go on with chest compression. Push. Push. Push.“ (repeating) |
| „Stop chest compression. Ventilation. Ventilation.“ |
| “Stop HWL.” |
|  |
|  |
| “Don`t touch the patient. Heart rhythm is being analyzed. Please wait.” (repeating) |
| “Shock recommended.” |
| “Don`t touch the patient. Push the red |
| blinking button for shock administration.” |
| “Shock applied.” |
| “The patient can be touched now.” |

All voice prompts were translated in English language. § Voice prompts which are given when pressing the info button (Philips HS1 only).
All voice prompts were translated in English language.
